# Quarterly Report (No. 2,) of the Medical and Surgical Practice of the St. George's and St. James's General Dispensary, 14, Old Burlington-Street; from October 13, 1822, to January 12, 1823, Inclusive

**Published:** 1823-02

**Authors:** George Gregory

**Affiliations:** Senior Physician to the Dispensary, &c. &c.


### STATISTICAL MEDICINE.

ART. I.—*Quarterly Report (No. 2,) of the Medical and Surgical Practice of the St. George's and St. James's General Dispensary,* 14, *Old Burlington-street; from October* 13, 1822, *to January* 12, 1823, *inclusive.* By GEORGE GREGORY, M.D. Senior Physician to the Dispensary, &c. &c.

| | | | |
|---|---|---|---|
| Acute Constitutional Affections | Fever, Common Continued | 45 | |
| | —— Remittent, of Children | 21 | |
| | —— Bilious | 1 | |
| | Variola | 2 | |
| | Measles | 5 | |
| | Cynanche Maligna | 7 | |
| | —— Parotidæâ | 1 | |
| | Erysipelas | 2 | |
| | Rheumatism, Acute | 2 | |
| | —— Subacute and Chronic | 18 | |
| | Gout | 1 | —105 |
| Acute and Chronic Diseases of the Head and Nervous System | Hydrocephalus | 4 | |
| | Pain of the Head and Giddiness | 6 | |
| | Palsy | 3 | |
| | Mania | 1 | |
| | Epilepsy | 3 | |
| | Neuralgia | 2 | — 19 |
| Acute and Chronic Diseases of the Thorax | Pneumonia | 12 | |
| | Subacute and Chronic Bronchitis | 64 | |
| | Catarrh | 5 | |
| | Hæmoptysis and Phthisis | 14 | |
| | Pertussis | 12 | |
| | Affections of the Heart | 5 | |
| | Pain of the Side | 5 | —117 |
| Diseases of the Chylopoietic Viscera | Dyspepsia | 54 | |
| | Hæmatemesis | 1 | |
| | Constipation | 3 | |
| | Diarrhœa and Dysentery | 6 | |
| | Jaundice | 4 | |
| | Chronic Hepatitis | 4 | |
| | Diseases of the Rectum | 5 | — 77 |
| Diseases of the Urinary System | Nephritis | 1 | |
| | Gravel | 1 | |
| | Retention of Urine | 2 | |
| | Disease of the Kidney | 1 | |
| | Affection of the Bladder | 1 | |
| | Stricture of the Urethra | 1 | — 7 |
| Diseases of the Uterine System | Irritation from Pregnancy | 1 | |
| | Menorrhagia | 3 | |
| | Leucorrhœa | 3 | |
| | Amenorrhœa | 3 | |
| | Dysmenorrhœa | 1 | |
| | Hysteria | 4 | |
| | Diseased Uterus | 1 | — 16 |
| Chronic Constitutional Affections | Scrofula | 5 | |
| | Cachexia | 1 | |
| | General Debility | 3 | |
| | Climacteric | 1 | |
| | Hypochondriasis | 4 | |
| | Plethora | 1 | |
| | Dropsy | 3 | — 18 |

| | | | |
|---|---|---|---|
| Chronic Diseases of the Skin | Psora | 4 | |
| | Psoriasis | 6 | |
| | Porrigo | 5 | |
| | Prurigo | 2 | |
| | Urticaria | 2 | |
| | Noli me Tangere | 1 | |
| | Eruptions not well characterized | 5 | 25 |
| Syphilitic Affections | Syphilis | 4 | |
| | Ulcers of the Penis, and Bubo | 11 | |
| | Inflamed Testicle | 3 | |
| | Sarcocele | 1 | |
| | Nodes of the Shin | 2 | |
| | Ulcerated Sore Throat | 5 | |
| | Gonorrhœa | 8 | 34 |
| Diseases strictly Local | Fractures and Dislocations | 5 | |
| | Bruises, Sprains, and slight Injuries | 34 | |
| | Burns and Scalds | 5 | |
| | Sore Legs | 13 | |
| | Abscess of the Breast | 4 | |
| | Phlegmons, Abscesses, and Ulcers, in other parts | 27 | |
| | Ophthalmia | 7 | |
| | Rupture | 5 | |
| | Ozœna | 1 | |
| | Deafness | 1 | 102 |
| | Total | | 520 |

The admissions during the last quarter have fallen short of those of the preceding, but they still exceed the average of ordinary seasons,—viz. 500. By comparing the present with the preceding report, (vol. xlviii. p. 443,) it will be observed that a remarkable change has taken place in the character of the diseases. Thoracic affections, which in the preceding quarter only amounted to one-ninth, now constitute above a fifth part of the whole. In fact, the extent, severity, and obstinacy, of bronchial affections during the last two months, has been the peculiar feature of the medical history of the period. The extreme severity of the weather most satisfactorily explains it.

The fatal cases occurring during the quarter have amounted to eighteen; of whom twelve were admitted in the same period, and six in the preceding. They may be arranged in the following manner:—Fever, one; Encephalic Disease, three; Thoracic Disease, ten; Abdominal Disease, two; Diseased Hip, of long standing, one; Tumor of the Pharynx, one. Permission to examine the body was given, wherever the peculiar circumstances of the case rendered it adviseable to ask it, and the following cases are selected as having afforded some instruction.

1. An infant, eight months old, died after an illness of ten days: the chief symptoms being excessive dyspnœa, severe hard cough, without expectoration, very rapid and hard pulse, with great paleness and anxiety of countenance. On dissection, the lungs, bronchial tubes, and heart, appeared *perfectly healthy.* Some serum was effused upon the surface of the brain. The brain remarkably soft. No water in the ventricles. Abdominal viscera quite sound.

2. In a young man, twenty-five years of age, reported in the previous quarter as labouring under pneumonia, the pericardium was found covered, on its inner surface, with recent coagulable lymph, to a most uncommon extent. All trace of *cavity* was lost. There were no marks of any affection of the pleura. The disease had come on subsequent to attacks of acute rheumatism. The urgent symptom during life was a sense of weight, or *tightness*, at the epigastrium, preventing breathing.

3. Richard Barrell, æt. fifty-three, had been under treatment for two months for very anomalous symptoms, referred partly to the lungs, and partly to the liver and stomach. The peculiar symptom was excessive tenderness at the epigastrium, with an obstinately loaded tongue, and a pulse irregular and hardly perceptible. On dissection, the disease was found to consist in ossification of the pleura of the right side, which had extended over a very large portion of the membrane. The lungs were loaded with a watery secretion. Above an ounce of pure pus was found in the cavity of the pericardium. The liver and abdominal viscera exhibited no appearance of disease.

4. The case of fungous growth in the pharynx occasioning the death of the patient, occurred to Mr. Jeffreys, senior surgeon of the institution. A woman, forty-two years of age, had had "a *tight swallow*" from infancy, and her disorder had been considered to be stricture of the œsophagus. She was admitted on the 31st of October, labouring under the usual symptoms arising from permanent obstruction of the œsophagus: she sunk, and died on the 12th of December. On inspecting the body after death, a fungous tumor was found filling up the lower portion of the cavity of the pharynx. It took its origin by a narrow neck from the mucous membrane covering the back part of the cricoid cartilage, and completely blocked up the entrance of the œsophagus. It had a soft fleshy structure, and was about the size of a large walnut. The preparation is now in the museum of the College of Surgeons.

5. To these cases I may add another, which occurred to me a few days ago. A man of very corpulent habit, in perfect health, was suddenly seized, on the morning of the 11th of December, with an indescribable sense of weight, or suffocation, at the pit of the stomach. It recurred in about an hour afterwards, when he dropt down dead. On opening the body, a great accumulation of fat was found in the omentum and mesentery. No disease could be traced in any of the thoracic viscera. The great sinuses of the brain appeared loaded with blood, and there was considerable effusion of serum, both on its surface and within the ventricles. The abdominal viscera were healthy.

